# The potential threats and benefits of nephron-sparing surgery can be predicted by any available score system

**DOI:** 10.1590/S1677-5538.IBJU.2021.0424.1

**Published:** 2021-12-21

**Authors:** Anuar Ibrahim Mitre

**Affiliations:** 1 Universidade de São Paulo Faculdade de Medicina Hospital das Clínicas São Paulo SP Brasil Divisão de Urologia, Hospital das Clínicas da Faculdade de Medicina da Universidade de São Paulo – FMUSP, São Paulo, SP, Brasil

## COMMENT

The article Practical Evaluation of the R.E.N.A.L. Score System in 150 Laparoscopic Nephron Sparing Surgeries seems to be a revisit topic however it is a very well written paper that update almost all aspects of laparoscopic partial nephrectomy (1). Really emphasis the importance of using a score system. The value of nephrometric scores as an important tool is for long time very well stablished. It should be always employed to standardize in less subjective possible different renal tumors as well as predict the risks for a partial nephrectomy and its complications. It became more relevant in recent years since surgeons gained more expertise and consequently increased indication for more challenging nephron sparing surgeries. The smaller tumors may be followed, treated by thermal ablation, or even removed without vascular pedicle clamping.

I missed the reason to use R.E.N.A.L. score and not some other. Is it just a preference of authors or may be one of the best? Anyway, the potential threats and benefits of nephron-sparing surgery can be predicted by any available score system (2).

Nephrometric score based on 3D modeling (3D nephrometry score) may have some role preparing better the surgeon to foresee the difficulties than 2D scores (3).

Within the scope of the article, another issue that should have been discussed is thermal percutaneous ablation for treatment of stage T1a renal cell carcinoma. Surgery even considered the state of art interventional treatment, the percutaneous thermoablation of renal tumor of less than 3cm is as effective as surgery in terms of preservation of renal function, with lower invasiveness and risks, with similar oncological effectiveness (4).

In Brazil surgical robots are very expensive which limits its acquisition and maintenance in public hospitals of reference. However, despite not having robot the group treated the patients using minimally invasive surgery. Laparoscopic partial nephrectomy for long time demonstrated results comparable to open surgery and robotic surgery (5). Nevertheless, some groups point out better results in robotic partial nephrectomy (6).

Finally, I congratulate the authors for this worthy article, and I recommend its reading.
